# Long-term (up to 16 months) health-related quality of life after adjuvant tailored dose-dense chemotherapy vs. standard three-weekly chemotherapy in women with high-risk early breast cancer

**DOI:** 10.1007/s10549-020-05602-9

**Published:** 2020-03-31

**Authors:** Yvonne Brandberg, Hemming Johansson, Mats Hellström, Michael Gnant, Volker Möbus, Richard Greil, Theodoros Foukakis, Jonas Bergh, the Swedish Breast Cancer Group, the Austrian Breast, Colorectal Cancer Study Group, the German Breast Cancer Group

**Affiliations:** 1 Department of Oncology-Pathology, Karolinska Institutet, Stockholm, Sweden; 2 Department of Oncology, Karolinska University Hospital, Stockholm, Sweden; 3 Department of Surgery, Medical University of Vienna, Vienna, Austria; 4 Department of Gynecology and Obstetrics, Goethe University Frankfurt/M, Frankfurt, Germany; 5 Medical Department, Medical University Hospital, Salzburg, Austria; 6 Department of Oncology-Pathology, Karolinska Institutet, BioClinicum J5:17, Karolinska University Hospital, Solna, 171 64 Stockholm, Sweden

**Keywords:** Breast cancer, Adjuvant, Dose dense, Health-related quality of life, Randomized trial

## Abstract

**Purpose:**

To prospectively compare HRQoL effects of two modern adjuvant chemotherapy breast cancer treatment regimens at six time-points up to 16 months after random assignment.

**Methods:**

The open-label, randomized, Phase 3 “Panther trial” was conducted between February 2007 and September 2011. 760 women, aged 65 years and younger, after surgery for non-metastatic node-positive or high-risk node-negative breast cancer were randomized 1:1 to the experimental group (four cycles of tailored and dose-dense adjuvant epirubicin and cyclophosphamide/2 weeks followed by four cycles of tailored dose-dense docetaxel/2 weeks) or standard group (three cycles of fluorouracil and epirubicin-cyclophosphamide/3 weeks followed by three cycles of docetaxel/3 weeks). HRQoL was assessed at all Swedish centres using EORTC QLQ-C30 and EORTC QLQ-BR23 at six points during 16 months before randomization.

**Results:**

Response rates to questionnaires were highest at baseline 728/780 (93%) and lowest 16 months after randomization, 557/750 (74%). HRQoL declined during treatment in both groups. At the end of treatment, the experimental group reported statistically significantly lower HRQoL (*P* < 0.001) than the standard group on global health status, physical functioning, role functioning, social functioning, fatigue, sexual functioning, and systemic therapy effects. No differences were found for emotional functioning, body image, and arm and breast symptoms. There were no statistically significant differences between the groups at the first follow-up and at subsequent assessments. HRQoL levels at the 16-month follow-up were similar to baseline values.

**Conclusions:**

Negative HRQoL impact of the dose-dense and tailored strategy appears to be prominent during treatment, but HRQoL recover once treatment ends.

**Trial Registration:**

clinicaltrials.gov Identifier: NCT00798070; isrctn.org Identifier: ISRCTN39017665.

## Introduction

Adjuvant chemotherapy increases survival in early breast cancer [1, 2]. The “Panther trial” was an open-label, randomized, multi-centre Phase 3 study, conducted in 86 study sites in Sweden, Germany, and Austria [3]. The aim of the trial was to determine whether tailored dose-dense adjuvant chemotherapy improves the outcomes of early breast cancer, compared with a conventional three-weekly chemotherapy schedule. After a median follow-up of 5.3 years there were 151 relapses or deaths due to breast cancer in the standard group and 118 in the experimental group (HR 0.79; 95% CI 0.61–1.01; log-rank *P* = 0.06). In addition, the experimental group had significantly better event-free survival than the standard group (HR 0.79; 95% CI 0.63–0.99; *P* = 0.04). Furthermore, increasing the dose density of adjuvant chemotherapy by more frequent administration is safe and results in fewer disease recurrences and fewer deaths from breast cancer as shown in a recent meta-analysis including 15,212 women in 15 randomized trials [4]. Based on these results dose-dense administration of chemotherapy will be further used.

Health-related quality of life in women undergoing these regiments has, to our knowledge, not been reported previously. Chemotherapy in conventional doses has been reported to have a negative impact on the patients’ health-related quality of life (HRQoL) during treatment [5]. In a Cochrane review, comparing high-dose chemotherapy and autologous bone marrow or stem cell transplantation versus conventional chemotherapy, HRQoL was reported as a secondary end point [6]. The review concluded that women undergoing high-dose therapy reported significantly lower levels of HRQoL during and immediately after treatment, but that few statistically significant differences were found between the groups after 1 year. In a Scandinavian study, HRQoL was compared in 525 patients at eight points of assessment during the first year after random assignment to treatment with tailored fluorouracil, epirubicin, and cyclophosphamide (FEC) therapy for nine courses versus induction FEC therapy for three courses followed by high-dose chemotherapy with cyclophosphamide, thiotepa, and carboplatin (CTCb) supported by peripheral blood stem cells [7]. No differences in HRQoL were found between the treatment groups in that study. HRQoL decreased significantly in both groups during treatment, but increased to baseline levels at the one-year assessment point. In the ADEBAR trial, 1306 patients with breast cancer were randomized to either group EC-DOC (four cycles of epirubicin at and cyclophosphamide followed by four cycles of docetaxel) or group FEC 120 (dose-dense six cycles of epirubicin and 5-fluorouracil at with cyclophosphamide) [8]. HRQoL was assessed at baseline, before cycle 4 FEC and cycle 5 EC-DOC, 4 weeks after chemotherapy, and 6 weeks after radiotherapy, using EORTC QLQ-C30 and the breast cancer specific EORTC QLQ-BR23. HRQoL, defined by five pre-selected subscales (global QoL, physical functioning, nausea and vomiting, fatigue, and systemic therapy side effects) declined in both groups during treatment, more in the dose-dense group, but returned at the last assessment to levels above the ones found at baseline. Statistically significant differences were found, favouring the EC-DOC group, during and shortly after stopping the treatment concerning fatigue and chemotherapy-related side effects. The differences were not, however, of clinical significance. HRQoL results are important in the light of the newly published results of survival gain of dose-dense chemotherapy in high-risk early breast cancer [4]. We have not found any other published trial, assessing HRQoL in the dose-dense setting.

In the Panther trial, no statistically significant improvement in the primary end point, breast cancer relapse-free survival, was found for the tailored dose-dense therapy, although the tailored therapy led to statistical significant improvement in the secondary end point, event-free survival at 5 years of follow-up. HRQoL was a secondary outcome in the Panther trial and was found to be significantly worse for the tailored dose-dense group at the end of study treatment [3].

The primary aim of the present paper was to prospectively compare the HRQoL effects of the two treatment regimens up to 16 months after random assignment to adjuvant treatment with tailored dose-dense chemotherapy (Experimental group) versus three-weekly adjuvant chemotherapy (Standard group) in the Panther trial in Sweden. Special emphasis was put on the 8 months assessment at first follow-up.

## Patients and methods

### Patients

Women, aged 18 to 65 years, with histologically confirmed, completely resected invasive primary breast cancer that was axillary node positive or high-risk node negative without distant metastases were eligible. Inclusion and exclusion criteria have been previously described in detail [3]. The present paper includes patients from the Swedish participating centres, as it was stated in the protocol that HRQoL was mandatory only in Sweden. The response rates to the questionnaires in Austria and Germany were sufficient until end of treatment, and these results have been published [3]. Response rates dropped, however, in a number of the Austrian and German study sites to levels below 40% due to administrative failure during follow-up after end of treatment. Therefore, we did not consider it suitable to include data from Austria and Germany in the analyses of HRQoL during follow-up.

### Treatment regimens

The patients were randomized to either four cycles of leukocyte nadir-based tailored and dose-dense adjuvant epirubicin (E) and cyclophosphamide (C) every two weeks, followed by four cycles of tailored and dose-dense adjuvant docetaxel every 2 weeks (Experimental group), or to standard-interval three cycles of fluorouracil/epirubicin/cyclophosphamide (FEC) every 3 weeks, followed by three cycles of docetaxel every 3 weeks (Standard group).

Patients with human epidermal growth factor receptor 2 (HER2) positive disease received one year of adjuvant trastuzumab. All women with hormone receptor positive disease were given tamoxifen or aromatase inhibitors for at least 5 years, starting after the end of chemotherapy. The women were followed up with clinical visits, haematological, and biochemical tests as previously described [3].

### Points of assessment

HRQoL was assessed at six points: (1) baseline (before randomization), (2) 2 months (during treatment), (3) 4 months (end of treatment), (4) 8 months (1st follow-up), (5) 12 months (2nd follow-up), and (6) 16 months (3rd follow-up). The main assessment point in the present paper was at 8 months after randomization, at the first follow-up about four months after the end of treatment to examine HRQoL during recovery after the end of chemotherapy.

### Procedure

Patients were informed orally and in writing about the HRQoL study before inclusion into the clinical study. The physician entering the patient into the clinical study handed the first questionnaire to the patient. This questionnaire was completed before information was conveyed about the treatment regimen to which the patient had been randomized. Subsequent questionnaires were administered by study nurses and forwarded to the study coordinator at the Clinical Trials Unit, Department of Oncology, Karolinska University Hospital, Stockholm. Data were imputed into the study database. HRQoL was no longer assessed after relapse of the disease.

### Instruments

European Organization for Research and Treatment of Cancer Quality of Life Questionnaire-C30, version 3.0 (EORTC QLQ-C30) includes nine multi-item scales and six single item variables [9]. Five functional scales consist of physical-, role-, emotional-, social-, and cognitive functioning. Fatigue, nausea/vomiting, and pain comprise the three multi-item symptom scales. Additional symptoms are assessed by single items: dyspnoea, sleep disturbances, appetite loss, constipation, and diarrhoea. One single item scale concern financial problems related to disease and treatment. Most items are responded to on a four-point scale ranging from 1 (not at all) to 4 (very much). The two items assessing global health and overall quality of life are responded to in seven categories ranging from 1 (very poor) to 7 (excellent).

The EORTC QLQ Breast Cancer Module (EORTC QLQ-BR23) is a breast cancer specific questionnaire, developed for use among patients varying in breast cancer disease stage and treatment modality [10]. It comprises 23 items, divided into four functioning scales: body image, sexual functioning, sexual satisfaction/enjoyment, and future perspective, and four symptom scales: systemic therapy side effects, breast symptoms, arm symptoms, and being upset by hair loss. The items are responded to in the same four categories as most items in the EORTC QLQ-C30.

The selection of subscales for analysis was based on a previous randomized trial [7]. Scales where patients given tailored FEC reported the highest levels of problems in that trial were selected. Thus, the following variables from the EORTC QLQ-C30 were chosen: physical functioning, role functioning, emotional functioning, social functioning, fatigue, and global quality of life. The following EORTC BR-23 variables were included: body image, sexual functioning, systemic therapy side effects, breast symptoms, and arm symptoms. The variables “future perspective”, “sexual satisfaction/enjoyment”, and “upset by hairloss” were excluded, as these problems were not expected to show differences between the randomization groups.

## Statistical considerations

### Sample size and power

No special power considerations regarding HRQoL were stated in the study protocol. The power calculation in the Panther trial was based on the primary end point, breast cancer recurrence-free survival.

Data for the EORTC QLQ-C30/BR23 were scored according to the EORTC QLQ-C30 scoring manual [11]. All scales and single items were linearly transformed to 0–100 scales where

- a high score for a symptom scale represents a high level of symptoms or problems
- a high score for a functional scale represents a high level of functioning
- a high score for the global health status/QoL represents high quality of life.

In the interpretation of the EORTC QLQ-C30 and BR-23 scores, a difference of ≥ 5 points on the 0–100 scales was considered as clinically important. Differences of 5–9 points were small, those of 10–19 moderate, and ≥ 20 large [12].

### Response rates and missing data

In order to investigate predictors for missing data at baseline (missing/non-missing), the association between this outcome and the clinical factors age (< 50/≥ 50), Grade (I/II/II), positive nodes (0/1–3/4–9/> 9), tumour size (0–20/21–50/> 50), oestrogen receptor (ER) and progesterone receptor (positive/negative), type of surgery (mastectomy/breast-conserving surgery), allocated treatment (Experimental group/Standard group) were tested using the Chi-square test for independence. Logistic regression was used to estimate the effect of the clinical factors, and HRQoL scores, on the risk of being missing at the next assessment point for each scale.

### Main analysis and statistical model

The main analysis was based on all available data for Sweden. In order to check the robustness of the main results, both single and multiple imputations were performed. Single imputation was performed by imputing the lowest possible scale score 0, or the highest possible score 100 for all missing data. Multiple imputation—assuming missing at random (MAR) was performed by using multivariate normal regression with five imputations added, using the predictors age, positive nodes, ER, PgR, type of surgery, and country. For these predictors, data were available for all patients. Imputation was done separately for each randomization group.

The effects of treatment on the different HRQoL scales were estimated using linear mixed models, with an unstructured covariance matrix including country, treatment, time, and the interaction between treatment and time. For each scale, all scores (including baseline) over time were used as the dependent outcome in the models. Tests for differences in treatment effect at eight months were obtained by linear combinations of the treatments, time, and the interaction estimates at that point. Results from the regression models are presented as mean differences, 99% confidence intervals, and Wald *P*-values. Graphically, results from the study are presented for the eleven pre-selected scales as scale means (SDs) at baseline by treatment and mean scale profiles by time and treatment.

Due to multiple testing, the level of statistical significance was set to 0.01 to guard against Type I errors. All analyses were performed according to the “intention-to-treat” principle.

The Ethics Review Boards approved the study with jurisdiction for all the participating sites.

## Results

A total of 780 patients were included in the Panther trial in Sweden. Baseline clinical and demographic data according to randomization group are presented in Table 1. Response rate was defined as the number of patients with at least one of the 23 subscales completed, divided by the patients at risk. Rates of responses to questionnaires among event-free patients were 728/780 (93%) at baseline, 682/780 (87%) 2 months after randomization during treatment, 686/778 (88%) 4 months after randomization at the end of treatment, 639/772 (83%) 8 months after randomization at the first follow-up, 577/757 (76%) 12 months after randomization at the second follow-up, and 557/750 (74%) at 18 months after randomization at the third follow-up. There were no differences in response rates between the randomizations groups (data not shown).

**Table 1:** Baseline characteristics of the 780 Swedish patients according to randomization group

Characteristic	Treatment group, no. (%)	
	Experimental group (tailored dose-dense chemotherapy) *n* = 384	Standard group (standard chemotherapy) *n* = 396
Age, median [min–max]	51.9 [24.7–64.8]	50.0 [21.4–65.9]
Type of surgery		
Mastectomy	234 (61)	237 (60)
Breast-conserving surgery	150 (39)	150 (40)
Tumour size, cm		
≤ 2	158 (41)	158 (40)
> 2 to 5	196 (51)	215 (54)
> 5	30 (8)	23 (6)
Positive nodes, no.		
0	22 (6)	17 (4)
1–3	236 (62)	233 (59)
4–9	87 (23)	110 (28)
> 9	39 (10)	36 (9)
Tumour grade		
1	29 (8)	24 (6)
2	151 (39)	171 (43)
3	201 (52)	201 (51)
Missing	3 (1)	0 (0)
Hormone receptor status		
ER or PR positive	297 (77)	298 (75)
ER and PR negative	86 (23)	98 (25)
Missing	1 (0)	0 (0)
HER2 status		
Negative	313 (82)	312 (79)
Positive	71 (19)	84 (21)
Ki-67 positive cells, IHC		
≤20	150 (39)	155 (39)
> 20	197 (51)	200 (51)
Missing	37 (10)	41 (10)

### HRQoL differences between the randomization groups over time

**Fig. 1:**
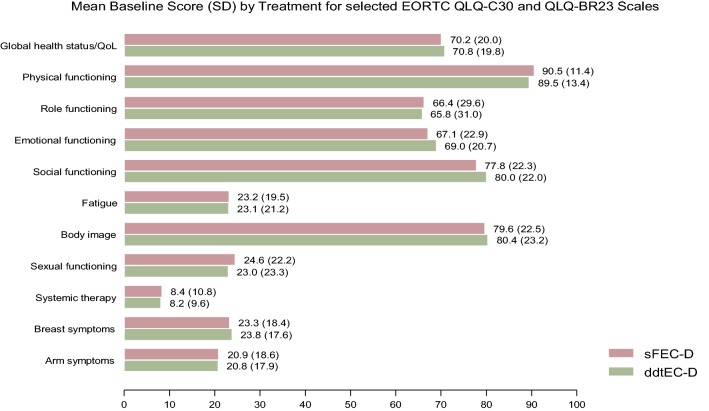
Mean baseline score (SD) for selected EORTC QLQ-C30 and EORTC QLQ-BR23 scales

**Fig. 2:**
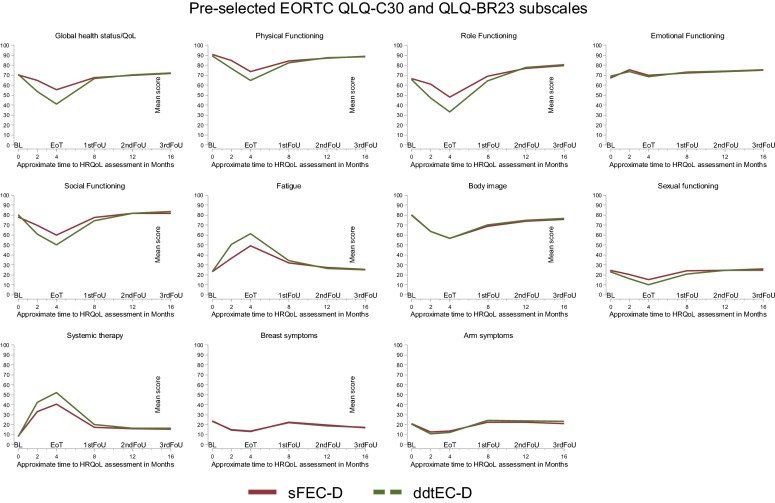
Mean scale scores for pre-selected EORTC QLQ-C30 and EORTC QLQ-BR23 scales at six points of assessment

There were no statistically significant differences in HRQoL between the randomization groups at baseline, Fig. 1. At the end of treatment, 4 months after randomization, the Experimental group (tddEC-D) scored statistically significantly lower than the Standard group (FEC-D) on five of the six functional scales, with the exception of emotional functioning where no difference was found, Table 2. The Experimental group also reported a statistically significant lower level of sexual functioning and a higher level of systemic therapy side effects compared to the Standard group, but no differences were found for body image, and arm and breast problems between the groups. All of the statistical significant differences were of clinical relevance, Table 2. At the 8 months assessment, at the first follow-up visit, no differences were however found between the randomization groups, with the exception of a “small” clinical difference for role functioning. There were no differences between the randomization groups at the subsequent assessment points up to sixteen months after randomization, Fig. 2.

**Table 2:** Mean values, standard deviations (SD), mean difference (MD), 99% confidence intervals (CI) at the end of treatment and at the first follow-up visit according to treatment group

Pre-selected scales	At end of treatment (4 months)				At first follow-up visit (8 months)				Test for interaction^‡^
EORTC subscales	Experimental group Mean (SD)	Standard group Mean (SD)	MD^#^ (99% CI)	*P*	Experimental group Mean (SD)	Standard group Mean (SD)	MD^#^ (99% CI)	*P*	*P*
EORTC QLQ-C30									
Global health status	41.0 (21.4)	55.2 (21.0)	− 14 (− 18 to − 10)^M^	< 0.001	66.6 (18.8)	67.6 (19.7)	− 1 (− 5 to 3)	0.570	< 0.001
Physical functioning	64.9 (21.5)	73.6 (18.9)	− 9 (− 12 to − 6)^S^	< 0.001	82.2 (16.6)	84.4 (14.6)	− 3 (− 6 to 1)	0.031	< 0.001
Role functioning	33.2 (29.2)	47.8 (30.9)	− 15 (− 20 to − 9)^M^	< 0.001	64.3 (29.6)	69.1 (28.3)	− 5 (− 10 to 1)^S^	0.028	< 0.001
Emotional functioning	68.3 (22.3)	69.9 (22.6)	− 2 (− 6 to 3)	0.307	73.3 (21.4)	72.0 (23.6)	2 (− 3 to 6)	0.353	0.055
Social functioning	50.1 (27.9)	59.9 (25.4)	− 10 (− 15 to − 5)^M^	< 0.001	74.4 (25.0)	77.4 (23.5)	− 3 (− 7 to 2)	0.160	< 0.001
Fatigue	61.3 (25.6)	49.1 (25.3)	12 (8 to 17)^M^	< 0.001	34.4 (23.1)	32.0 (22.7)	3 (− 2 to 7)	0.157	< 0.001
EORTC QLQ− BR23									
Body image	56.7 (28.8)	56.7 (29.5)	0 (− 5 to 5)	0.896	70.1 (25.5)	68.9 (28.0)	2 (− 3 to 7)	0.414	0.884
Sexual functioning	10.2 (15.9)	15.1 (18.7)	− 5 (− 9 to − 1)^S^	0.001	20.9 (21.3)	24.3 (21.1)	− 3 (− 7 to 1)	0.045	0.057
Systemic therapy	52.2 (18.2)	40.7 (19.2)	12 (9 to 14)^M^	< 0.001	19.9 (14.1)	17.2 (13.9)	3 (0 to 5)	0.024	< 0.001
Breast symptoms	13.3 (14.9)	13.5 (15.0)	0 (− 4 to 3)	0.785	22.7 (19.7)	21.8 (19.1)	0 (− 3 to 4)	0.762	0.904
Arm symptoms	12.0 (16.4)	13.8 (16.5)	− 2 (− 5 to 2)	0.194	24.2 (21.2)	22.5 (19.8)	2 (− 2 to 6)	0.182	0.077

^#^Mean difference and 99% confidence intervals estimated using linear mixed effects model with a random intercept and a random slope

^‡^A significant treatment × time interaction indicates different effects of treatment over time

*S* Small clinical difference, *M* Moderate clinical difference (Osoba 1998)

## Discussion

HRQoL in a tailored and dose-dense schedule was compared with a standard three-weekly schedule of adjuvant chemotherapy in breast cancer patients. HRQoL decreased during treatment in both groups, but more in the Experimental group as compared to standard treatment. There were, however, no differences in HRQoL between the randomization groups at the first follow-up visit or during follow-up to the third visit, 16 months after randomization. The results indicate that the women recovered once treatment had stopped, congruent with the findings of other studies comparing high-dose chemotherapy with standard treatment [6, 7, 14].

During adjuvant treatment, in line with the findings of previous research, HRQoL was negatively impacted in several areas in both groups [5, 8, 14]. As the decline in HRQoL appeared during treatment, it is likely this decline was related to the side effects of the treatments. A study comparing HRQoL between different doses of tailored chemotherapy found that patients who received higher doses, based on the tailored dosing strategy, did not seem to have worse HRQoL than those who had lower doses [15]. In the present study, the regimen regarding tailoring of doses were similar as in our previous study [7]. In that study, the levels of HRQoL at the 16 weeks assessment (corresponding to the 4 months assessment point) for the tailored dose group were similar to the Experimental group in the present study concerning physical and emotional functioning. For role and social functioning, as well as for global quality of life, the patients in the present study appeared to have lower levels, indicating more problems. In addition, they tended to report a higher level of fatigue as compared to the patients receiving tailored therapy in the previous study. These results indicate that the dose-dense therapy had an additional effect on HRQoL, besides the effects caused by the tailored therapy.

In the ADEBAR trial, HRQoL values at the last assessment point, 6 weeks after radiotherapy, appeared to be better than baseline values, indicating that the patients also in that study recovered once treatment was terminated [8]. The response rate to the questionnaires at the last assessment was, however, low, 26%. It is reassuring that the levels of HRQoL found in the Experimental group at the eight months assessment were similar to the levels reported in the previous study for the tailored dose group at the 30 weeks assessment point, except for role functioning that appeared to be lower in the Experimental group in the present study.

Emotional functioning did not show change over time, and the levels up to the eight months assessments were similar to those found for the tailored dose group in a previous study comparing tailored dose chemotherapy with bone marrow transplantation [7]. In that study, the patients reported the lowest levels of emotional functioning at the time of randomization, and emotional functioning improved during the first year after randomization. Many patients express that the worst phase, emotionally, is after diagnosis before start of treatment, when they live in uncertainty and do not know what to expect. Once treatment has started, the patients feel that actions are taken against their cancer, and they are in the process of adaptation to their situation.

The strengths of the study are the large sample, the randomized design, the use of standardized validated questionnaires and the relatively high response rate in Sweden. The major weakness was that HRQoL was also assessed in Germany and Austria, although not mandatory. Subsequently, and due to administrative failures, the response rates in these countries were too low for consistent data analyses after the end of treatment. Thus, the present paper includes Swedish patients only.

## Conclusions

HRQoL levels expectedly declined during the treatment phase of adjuvant chemotherapy in breast cancer patients, more in the dose-dense group. Both groups’ HRQoL recovered to baseline levels found before commencing adjuvant chemotherapy, and no between-group differences were found at any time of follow-up after the end of treatment. Thus, the HRQoL impact of the dose-dense and tailored strategy appears to be prominent during treatment, but patients recover once treatment has ended.
